# A Box to Put the Baby in: UK Parent Perceptions of Two Baby Box Programmes Promoted for Infant Sleep

**DOI:** 10.3390/ijerph182111473

**Published:** 2021-10-31

**Authors:** Helen L. Ball, Catherine E. Taylor, Cassandra M. Yuill

**Affiliations:** 1 Department of Anthropology, Durham University, Durham DH1 3LE, UK; c.e.taylor@durham.ac.uk; 2 School of Health Sciences, City, University of London, London EC1V 0HB, UK; cassandra.yuill.2@city.ac.uk

**Keywords:** safer sleep interventions, sudden unexpected infant death (SUDI), Pēpi-Pods, baby boxes, SIDS prevention

## Abstract

Between 2016 and 2019, two different infant sleeping-box interventions were implemented in England: (1) shallow polypropylene baby boxes were distributed via a feasibility study to families with Sudden Infant Death Syndrome (SIDS) risk factors; and (2) a commercial–health system partnership scheme distributed cardboard baby boxes to new mothers in particular locations. We conducted parent evaluations of both interventions at the time of implementation. The views of 79 parents receiving polypropylene boxes and 77 parents receiving cardboard boxes were captured using online questionnaires and telephone interviews. Participants provided feedback on education received about using the box, their perception of the box design and materials, their experiences of using the box they received, and whether they would recommend it to others. Parents appreciated that both boxes provided a portable space to place their baby near them anywhere in the home, discouraging other riskier practices. The polypropylene box was rated more favourably regarding transparency, hygiene, and portability outside the home. A minority of parents found the idea of putting their baby in any box unappealing; however, younger mothers and smokers particularly appreciated the ability to safely co-sleep with their babies using the shallower box. Overall, the versatility of the polypropylene box scheme was more positively evaluated than the cardboard baby box scheme, which, stripped of its social value as part of a larger welfare provision, had minimal value for parents that received it.

## 1. Introduction

Prevention advice for Sudden Unexpected Infant Death (SUDI) encourages parents to ensure their baby sleeps in a safe space for every sleep. What constitutes a ‘safe space’ varies according to international (and sometimes regional) guidance, but most authorities emphasise placing babies supine on a flat surface that is clear of loose bedding and other suffocation or overheating hazards [1]. Although this guidance is offered under the assumption that all parents will have suitable safe places available for infant sleep, the lived realities of parenting can sometimes make compliance with idealised guidance unlikely or impossible [2,3,4,5,6]. In recognising that babies may sometimes be placed to sleep on unsuitable surfaces, or in hazardous contexts, novel interventions have been devised to provide parents with a safe (or safer) space in which their baby can sleep [7,8,9,10].

Two forms of boxes repurposed as infant sleep spaces became popular as infant sleep interventions in recent years. Firstly, small shallow polypropylene boxes (originally underbed storage boxes) fitted with mattresses and bedding were distributed to displaced New Zealand families in the aftermath of the 2011 Christchurch earthquake [8,9,10,11]. The success of this intervention led to the development of a customized box, bedding and distribution approach known as the Pēpi-Pod^®^ Programme (pēpi being a Maori term for baby). The box was increasingly used, always within the context of the programme, as a culturally-targeted safe sleep enabler for indigenous communities firstly in New Zealand, and subsequently Australia [12,13,14,15,16,17,18].

At the other end of the world, interest in cardboard baby boxes (similar to file storage boxes, again with a fitted mattress) was ignited following a UK news story about the Finnish maternity scheme where new parents can opt to receive a box full of baby supplies as part of the state-provided maternity package [19]. News reports erroneously claimed the cardboard baby boxes (which are equipped with a mattress and can be used as a portable sleep space) had contributed to Finland’s low infant mortality rate [20]. This resulted in a variety of copy-cat schemes around the world [21] including in England [9,22], Ecuador [23], Scotland [24], USA [25,26,27], and Zambia [28]. Regardless of the lack of evidence that cardboard baby boxes reduce SIDS [29,30], they have been widely promoted as SIDS prevention devices [31,32].

In the period 2016–2017, we undertook a feasibility study of the use of polypropylene baby boxes in the UK with families whose babies were potentially at increased risk for SIDS titled Let’s Talk About Sleep (LTAS)) [33]; and in the period 2018–2019, we undertook an evaluation of a commercial—NHS partnership scheme to implement cardboard baby boxes in England titled Baby Box Evaluation (BBE) [9]. Drawing upon data generated during these two projects, this paper compares the perceptions of UK parents to these two superficially similar interventions for providing babies with a safe place to sleep. In so doing, we aim to summarise the costs and benefits of these devices, better understand what makes such interventions acceptable/unacceptable to their intended recipients and consider whether one box is preferable as a baby sleep space over the other.

## 2. Materials and Methods

Data presented here are drawn from the intervention arm of the ‘Let’s Talk About Sleep’ (LTAS) feasibility study conducted in the period 2016–2017, and the Baby Box Evaluation (BBE) study conducted the period 2018–2019. Both studies were undertaken by staff of the Durham Infancy & Sleep Centre with approval from the University of Durham Research Ethics Committee; additionally, the LTAS study received approval from the National Health Service (NHS) Research Ethics Committee (ref: IRAS 184634) as participants were recruited in UK NHS settings. All participants provided written informed consent, and all identifiable data were handled according to General Data Protection Regulation (GDPR) principles.

**Figure 1:**
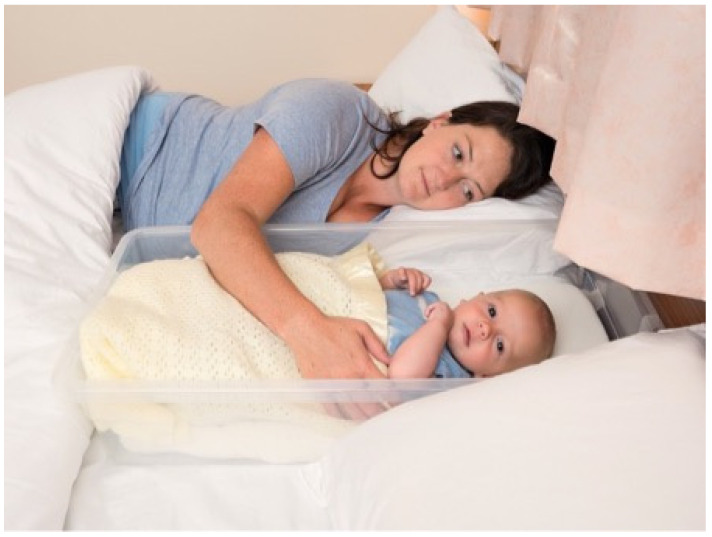
Mother and baby using polypropylene box (Reprinted with permission from Baby Sleep Information Source 2016 Rob Mank Photography).

**Figure 2:**
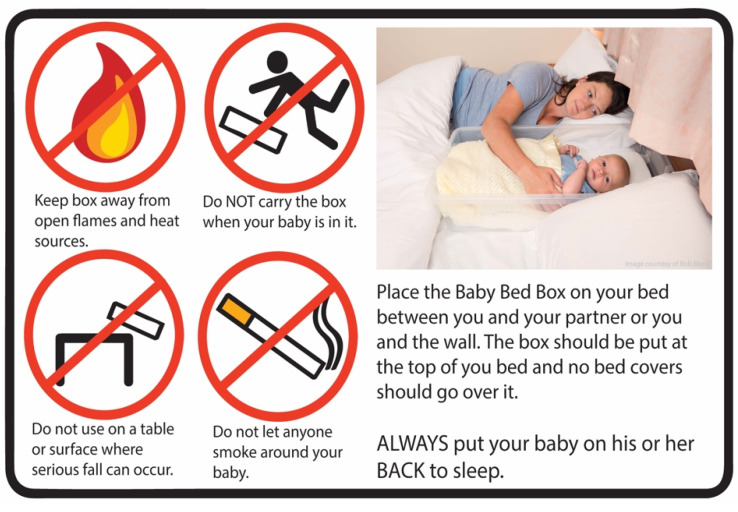
Polypropylene box safety sticker.

**Figure 3:**
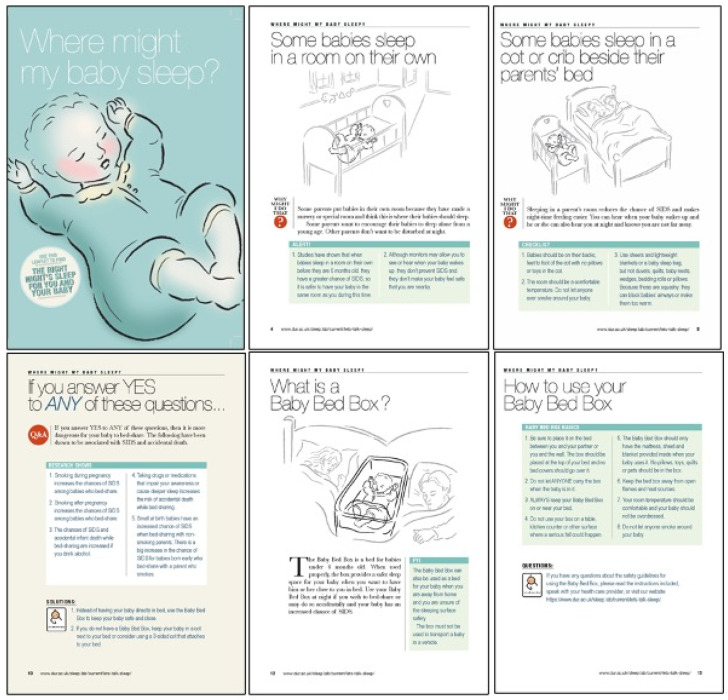
Polypropylene box information leaflet (pages 1, 4, 5, 10, 12, 13).

LTAS was a planned feasibility study that took place in two UK areas with high socioeconomic deprivation scores (Multiple Index of Deprivation) and high rates of maternal smoking: Sunderland in north-east England and Fife in south-east Scotland. In both locations, parents giving birth at local hospitals during the study period were invited by NHS staff to enrol in this study to receive an infant sleep box [33]. The box was designed as a locally-sourced facsimile of the New Zealand Pēpi-Pod^®^: a shallow polypropylene box (bisphenol and phthalate free) that was rigid, transparent, easy to clean, and long lasting (72.5 cm × 33.5 cm × 18 cm). A custom-sized mattress with a waterproof zipped cover compliant with British Safety Standards was included (see Figure 1). Locally-sourced fleece baby blankets and fitted cotton sheets were provided with the box (these varied from those developed for and supplied with the original Pēpi-Pod^®^), along with safety guidance (see Figure 2) and a professionally designed graphic and written leaflet of infant sleep safety information based on a previously published safer sleep educational intervention [34] (see Figure 3). Provision of educational material was a key feature of the original Pēpi-Pod^®^ Programme that we wished to replicate [35]. The box was presented to parents as a tool they could use for avoiding potentially hazardous co-sleeping such as on sofas or with a parent who was a smoker. Parents were asked to use the box as needed for a two-month period following their baby’s birth. Data on parents’ attitudes towards and experiences with the box were collected by research staff at the end of each month via an online questionnaire or via telephone interview according to participant preferences. Following the end of this study, boxes were collected by the research team so they would not be passed on without guidance on safe use.

**Figure 4:**
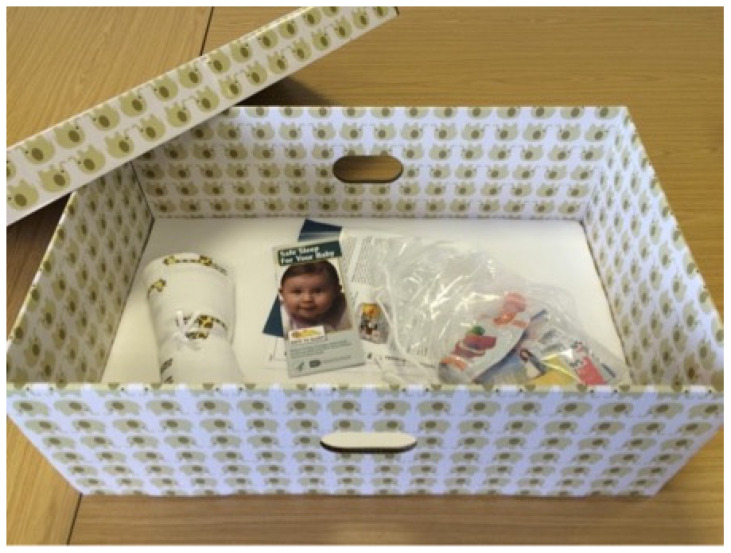
Cardboard baby box with free samples (photo by CM Yuill).

BBE was conducted opportunistically following the implementation of a flurry of cardboard baby box schemes in England between 2016 and 2019 that operated via an NHS-commercial partnership model [9]. These schemes were initially free for parents (later requiring a payment for delivery) and offered access to an online education platform and a baby box (65 cm × 40 cm × 28 cm) containing a mattress, sheet, and some infant-care products (see Figure 4). To obtain a box parents registered on the commercial box-provider’s website and consented to their data being captured. They then gained access to free educational videos and completed a multiple-choice quiz. Successful quiz completion led to a certificate for a baby box to be obtained from a participating hospital or health centre. Healthcare providers promoted the boxes to expectant parents, displaying posters, engaging with the media, and informing parents of the scheme. When the BBE project commenced 24 NHS facilities in England were offering baby boxes in seven of the nine English NHS regions (4 in Greater London, 3 in South East, 4 in West Midlands, 3 in North West, 2 in Yorkshire & Humber, 1 in East Midlands, and 4 in East of England). These programmes ceased when the US-based company providing the baby boxes went into liquidation in late 2019. Evaluation data were collected via an anonymous online survey hosted on a secure research platform, and shared by infant care organisations in England in newsletters and via social media in regions where baby box schemes were established. Parents/carers were eligible to participate if they resided in England and were familiar with a local baby box programme (whether they had received a box or not). Survey participation was voluntary, and no incentives were offered. The objectives were to capture parents’ attitudes towards and experiences with the NHS Trust-endorsed cardboard baby boxes.

## 3. Results

### 3.1. Participants

In the LTAS study, 79 expectant mothers (and fathers where present) were recruited to receive a polypropylene box (39 in Sunderland, 40 in Fife); all met the criteria of being at increased risk for SIDS due to parental smoking and/or young parental age. Follow-up data were obtained from 86% (68/79) of participants. Sociodemographic characteristics of the participants are shown in Table 1.

In the BBE study, seventy-seven (77) parents voluntarily completed the online survey —95% (73/77) of whom had been offered or applied for a cardboard baby box, and 89% (65/77) of whom had received one. All respondents were mothers of infants born between 2017 and 2019 residing in one of the English health districts where baby boxes were available. No other demographic information was requested from respondents to this online survey

### 3.2. Information Provision

Polypropylene box scheme: Overall, LTAS recipients were very positive about the education provided with the polypropylene box, feeling it offered much more information, particularly realistic and practical information than other information leaflets they had received. Several participants reported they knew nothing about infant sleep safety until they received this leaflet and that it was the only information they were given. Participants felt they had benefitted from the in-depth discussions around infant sleep safety and had enjoyed talking through the leaflet. One interviewee reported the leaflet was “handy and easy to understand” and that as her midwife explained every page, she gained a lot of information. In contrast, another commented that a midwife had simply shown her the leaflet and told her what it was about (not discussed it page by page), while a participant who picked up a box a few days after her baby was born commented “There was no leaflet or safety guidelines with it. Maybe they forgot them!”. Proportions of respondents reporting on the type of education provided is shown in Table 2.

Some participants experienced difficulties with literacy. This was reflected in comments expressing preference for visual and verbal information delivery. A narrated video of the leaflet content was available online and participants were provided with the link for this in their study pack. Multiple participants reported sharing the information with their partner and in some cases their wider family; however, one father stated that he was aware of his partner receiving the leaflet, but he did not read it himself, and she did not discuss it with him.

Cardboard box scheme: In the BBE, participants who intended to use the carboard box as a baby sleep space were asked if they were offered educational information, including how to use the box safely: proportions receiving each type of education are shown in Table 2; 6% received no educational information and 8% could not recall whether they had received any information about using the box they had been given. Respondents receiving information identified this as covering safe sleeping (80%), baby box safety (68%), and general infant care (43%). Some parents (15%) who accessed the online course reported they had not watched all the videos required to obtain their box, having found they could click on and off the recordings to get to the quiz, and then make multiple guesses at the answers to obtain their certificate for a box.

The content of the education videos provided by the English baby box schemes varied from one location to another. Local midwives and health visitors were recruited to write scripts and film videos for parents in their health district, and therefore emphasised different local needs and services (e.g., smoking cessation, car seat safety, maternal mental health). All programmes included a video from a US clinician on the safe use of the box, and where local information on infant sleep safety was not provided a US-video was sometimes substituted. Although parents were expected to only view the educational videos specific to their health district, it was possible for parents to view any of the videos from anywhere in the world. There did not appear to be any quality control over the educational information that was accessible by parents.

### 3.3. Using the Boxes

Polypropylene boxes: All but one LTAS participant reported they tried using the shallow polypropylene box and did so with varying degrees of success. Those who used it regularly were overwhelmingly in favour, with one commenting “I’m really sad he grew out of it”. This interviewee, who gave birth by Caesarean section, found using the box in bed made night-time feeding easier. Another responded that she really liked taking part in this study and using the box, which she mainly used at night in the bed, using her Moses basket downstairs during the day. Several participants indicated they used the box to avoid direct bed-sharing: “I smoke, so I don’t co-sleep (bed-share) because of SIDS. I just use the box.” This mother used the box in bed with her partner in a double bed with the box between them. Although the majority of LTAS participants indicated they used the box frequently one participant reported, “it was awkward at first because I’m a plus size woman” mentioning that if her bed was bigger, it might have been easier.

Participants who were single found the box worked well in a double bed, one commenting “I used it loads when he was first born”. This mother also reported that her friends and family liked the box because the baby settled easily in it, and that she used it when she stayed overnight at a friend’s house, putting it under the pram to transport it. Another also used it when staying overnight at a family member’s house rather than carrying a Moses basket and found it to be “very helpful”. She also described using it at home in a double bed, sometimes with her partner and other times with her daughter. She reported no issues with this arrangement and put the box against the wall if her daughter was in the bed, rather than putting it between them. Another participant and her partner reported they used the box while camping.

Participants were asked if they had used the box elsewhere than the parental bed. Half reported they had used it in the living room both during the day and in the evening. The box was either placed on the living room floor or on the sofa, and the box was also commonly used in other parts of the house during the daytime in order to keep infants close by while parents were carrying out other activities (i.e., cleaning, cooking or showering).

Participants who used the box less frequently offered less enthusiastic feedback. One reported she did not use the box very often, perceiving that her baby did not like the box and indicating it was difficult to use in the double bed she shared with her partner. Another reported she tried the box a couple of times, but that her baby “didn’t like it”. A third mother with a similar perception that her baby was happier in the Moses basket commented during her interview “now looking back I wished I had used it more because I think it’s good to have the baby close to you.” One couple had used the box for the first few weeks, but stopped because their weight would move the mattress on which the box was placed and the baby was “rolling around in it”. They also found it tricky to organise their duvet around the box. The father commented: “I like the concept. It’s headed in the right direction, but I think it should be tailored to single mums…I think it would be better for them because they have the space to be in bed with it.” Some participants expressed concerns over the safety of the box when used in the parental bed. These were worries about the adult bedding covering the box, the parent rolling onto the box, or the box accidently falling out of the bed. No participants reported experiencing these or any other adverse events.

The most common point of feedback provided on the box design was its transparency. Participants liked and felt reassured that they could see their baby easily through the side of the box when lying down and their baby could see them. There was general appreciation that the transparent box emulated a hospital bassinette. “I like it. It looks like what they have in the hospital after they’re born”; “You gave us the blankets and sheets which was really helpful”; “I thought the bedding was nice”; “I liked the bedding and clear plastic. It looks like the ones in the hospital.” However, one mother disagreed stating “I would definitely make it look less like the one in the hospital.” Suggestions for improvement included (a) to make the box deeper and rounder, (b) to add some padding on the sides, (c) to make it bigger to accommodate older babies, (d) to make it deeper, like the hospital versions and (e) devise a way to attach it to the bed-headboard, elevated above the mattress.

Cardboard boxes: Of the participants receiving a cardboard baby box, (88%, 57/65) were satisfied with the construction of the boxes, describing them as ‘sturdy’ and ‘solid’ while others commented on their durability if wet, or the unhygienic nature of porous cardboard. A total of 86% of parents that recalled receiving products in their box were either satisfied or somewhat satisfied with the box contents, while 14% expressed dissatisfaction, e.g., “I don’t believe that what was being advertised was delivered”.

Most survey respondents had placed their baby in the cardboard baby box for some part of the day or night, awake or asleep, at least once when the baby was 0–3 months of age (68%, 44/65). Of the 54% (35/65) of parents who regularly (at least once per week) used the box, only 17% (6/35) did so at night-time (baby awake or asleep). The majority of cardboard box-use (83%, 29/35) occurred during the day-time (baby awake or asleep); “Baby slept well in it during the day. It felt safe. I’m keeping it for my next baby.” Participants also reported using it in the bathroom so they could take a shower. “It is a safe place to put the baby if I need to do something while they are awake.” “Until he could pull up it was a great place to ‘store’ the baby after changing a nappy or if I needed free hands for a few minutes. The inside print design seemed to be very interesting for him.”

Approximately 23% (15/65) of box recipients had used the box when visiting friends/family or on holiday, the majority doing so regularly: “It is a useful space for baby to sleep when at my mother’s, I leave it at her house. Much safer and better than sleeping in a car seat.”

Reasons given for not using (or ceasing to use) the cardboard box included having another place to put the baby, not wanting to put the baby in a box, baby being unsettled in the box, and being unhappy with its construction or location: “Our first night home our daughter went in it and hated it”; “I think it’s not really suitable if you have older children as my son kept tripping over it and fell in it twice (baby wasn’t in it thankfully). I have met several mums saying same thing as it’s floor level and not safe to put on anything it’s not really ideal so I stopped using it for safety reasons.” Another respondent made a similar point that they had no surface safe enough to put the box on. Interestingly only one participant specifically mentioned that it is difficult to see the baby when they are inside the carboard box given the height and opacity of the sides: “I wanted to see my new-born constantly and take in his beautiful baby days. Also I wanted to constantly stare at him and check his breathing as most parents do—the box doesn’t allow this.”

Parents were asked whether they would recommend the cardboard baby box scheme to others: 75% (58/77) would, 16% (12/77) would not and 9% (7/77) had no opinion. Nineteen parents offered written comments highlighting evidence gaps around claims that the cardboard boxes prevented SIDS, highlighting more pressing areas of need for resource-investment, and noting lack of consistency in schemes and coverage, the need for clearer use instructions, and dissatisfaction with collection points/delivery charges. “I’m not sure what the point of them is to be honest. There is no evidence they reduce the incidence of SIDS. In this country it appears they are another way to advertise brands to new parents.” “I felt very frustrated after watching all the videos only then to find out the high price of postage and that the nearest collection point was over 40 miles away!”, “Waste of money. They should put the money used into breastfeeding support. Most people use as a storage box.”

## 4. Discussion

**Figure 5:**
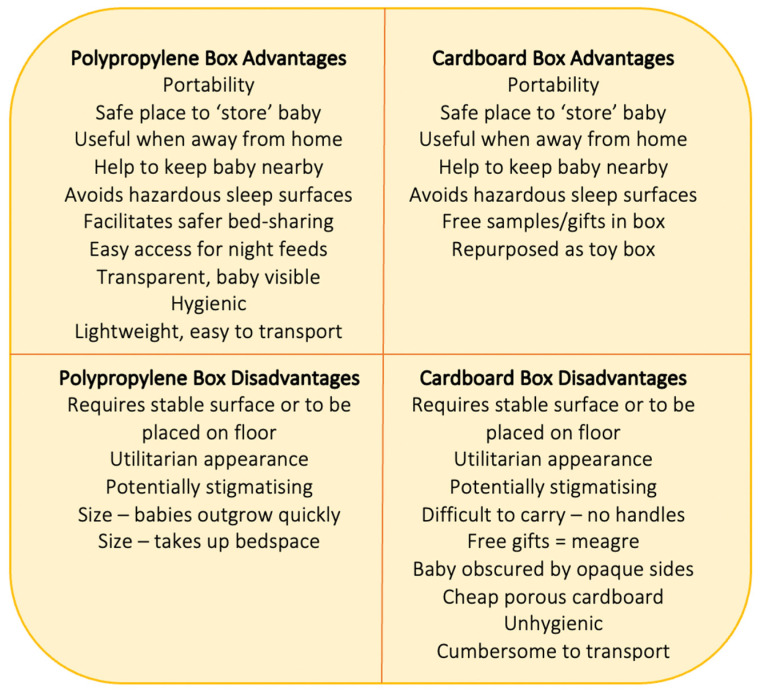
Advantages and disadvantages of baby box types identified by users.

The majority of parents who received boxes to put their babies in were positive about the general concept, and benefits were identified for each box type (see Figure 5). Benefits common to both boxes included portability inside and outside the home, and providing a confined and ‘safe’ place for babies to lie (or be ‘stored’) while awake or asleep. This was of benefit when parents/carers were engaged in activities such as showering or cooking, and when in a location that was not equipped to accommodate babies (e.g., homes of friends or relatives, camping). In these scenarios, both types of boxes facilitated parent–baby proximity during the daytime and discouraged riskier practices that might otherwise be used such as leaving the baby in a cot or crib in a room on their own, or placing babies in car seats or on sofas unattended.

Common negative perceptions of both box types included the need for a safe and stable surface to place the boxes on; some parents did not like placing their box on the floor, others simply did not like the idea of placing their baby in a utilitarian space, be it a cardboard box or a polypropylene box, also reported by US parents who found the idea of the box stigmatising [26]. It is relevant to note that the Pēpi-Pod^®^ sleep space implemented in New Zealand has been transformed from its origins as a utilitarian storage box to now have a dedicated design to address issues that were seen as barriers to safe sleep practices. Some parents were unhappy that for safety reasons the cardboard boxes were provided without lids or carrying handles. Few specific benefits were identified for cardboard baby boxes—some parents appreciated the free gifts that were provided with the box (although these were meagre), and that they could be used as a toy-box when no longer needed as a baby sleep space.

Overall, parents described more benefits and uses for the polypropylene boxes (see Figure 5). These included the ability to use the smaller shallower box on the parental bed at night to keep the baby close while reducing the hazards of bed-sharing with a smoker for instance. When using the box in this manner the transparency of the polypropylene box was also a benefit, allowing parents and babies to easily see one another while the shallow sides meant parents could touch their babies while lying next to them with easy access for night-time feeding. Parents also reported that the polypropylene boxes were hygienic, easy to clean and disinfect, and small enough to transport under a pushchair. Not mentioned by parents in this survey, but highlighted by health professionals [9] and other authors [29], is the potential for cardboard boxes to undermine breastfeeding by facilitating separation of mothers and babies, and making it difficult for mothers to see their babies’ early feeding cues, while the shallow polypropylene boxes had the potential to support breastfeeding by making night-time feeding easier by keeping mother and baby in close proximity both day and night.

While most parents considered the cardboard boxes to be robustly constructed, some thought them to be made of cheap flimsy cardboard that would collapse if wet. A key shortcoming of these boxes was the lack of ability to clean them, and their size made them difficult to transport without a car. Costs associated with the polypropylene boxes were primarily size related. Some parents wished they were bigger so they could be used for longer period before babies outgrew them; others felt they were too big for use on the parental bed between two adults, issues that were also reported by some parents who used the original Pēpi-Pods in Christchurch, New Zealand [8]. The latter was also a function of bed-size and the size of the parents. Single mothers and younger couples found the polypropylene box to fit easily on a double bed.

Both box types were accompanied by educational material for parents on the safe use of the box, and safer infant sleep generally. Following the expectations of the original Pēpi-Pod^®^ Programme, that the key function of the box is as an opportunity to convey infant safety information [13], the polypropylene boxes were designed with bespoke educational material in written and video formats. Practitioners were advised to discuss the information leaflet with mothers/parents before providing them with the box, and in the majority of cases this happened, but occasionally human error meant it was overlooked. Overall, in the LTAS study, the educational materials were highly praised as being practical and realistic, and providing helpful information.

Information accompanying the cardboard boxes was primarily in the form of videos that were locally written and narrated by health professionals, some of whom were unhappy at being portrayed as ‘SIDS experts’ by the commercial provider of the boxes [9]. That parents were able to skip through the videos and obtain a box without receiving safety education, and that videos made by the box-provider were not consistent with UK recommendations were major deficiencies of this scheme. Although English health professionals implemented the cardboard box schemes in good faith with the intention of using the boxes to engage families with pre and postnatal services, the NHS-commercial partnership was not sustainable nor effective in providing benefit to most parents and lacked the social value provided by the Finnish baby box programme [21]. Unless provided as a government run scheme with high quality baby supplies as in Finland or Scotland, embedded within a larger social welfare system, cardboard baby boxes alone are of little value, compared to the polypropylene boxes. The latter can be used in a versatile manner to promote sleep safety in a variety of contexts including as an in-bed sleep space for families where direct bed-sharing is contraindicated.

A missing component of both baby box programmes as implemented in the UK was the engagement of partners and wider family members in the interventions. While some participants shared information on the polypropylene boxes with their partners and family, the importance of doing so was not emphasised as a core feature of the intervention, despite being a key component (the Principle of Reciprocity) of the original New Zealand Pēpi-Pod^®^ Programme [8] and the subsequent partnership for the Queensland-based Pēpi-Pod^®^ Programme. In Queensland, strategies to embed core ethical principles when working in partnership with Aboriginal and Torres Strait Islander communities, including the development of mutually respectful partnerships and supporting capacity building within communities, complemented the original Progamme intent to uphold the Principle of Reciprocity [16].

In the cardboard baby box programmes, there was no incentive to involve partners in the decision to acquire a baby box, nor any suggestion that partners should be encouraged to watch the educational videos. Partners’ opinions were influential in some cases regarding the use of the polypropylene box in the parental bed and it would have been valuable to seek separate feedback from each parent. The views of practitioners regarding both box types are provided elsewhere [9,22,33], but in brief practitioners in general disliked cardboard baby boxes as cumbersome, unhygienic and barriers to responsive infant care. The polypropylene boxes were more acceptable to practitioners, but they expressed concerns about parents using them inappropriately (e.g., transporting the baby in the box).

Several limitations should be highlighted: although comparing the responses of parents in these two evaluation studies is useful for designing future safer sleep interventions, it is important to remember that these box-based interventions were conducted for different purposes, were adapted for use in the UK and therefore were not based on the methodologies of the programmes from which they derive, and the studies evaluating them were not designed to be compared—therefore, matching data are not available for all aspects of this comparison. The LTAS study was intended as a feasibility and acceptability precursor to a randomised trial of polypropylene boxes to reduce hazardous bedsharing practices among high-risk families. Participants were invited to take part if they met the criterion of being in a UK group known to be high-risk for SIDS (primarily smokers and younger mothers). The BBE study, on the other hand, sought to obtain opinions from parents who had received a cardboard baby box as part of an NHS-commercial partnership scheme. Although socio-demographic information was not captured from respondents to the BBE survey, given the skewed nature of respondents to online surveys [36,37], it is likely that they were generally older, more educated, and possibly better off financially than the LTAS participants, which may influence their perceptions of these schemes. It should also be remembered that at the time LTAS was conducted parents in the UK were unfamiliar with the concept of boxes as spaces to put their babies in and therefore several participants found the concept bizarre and unappealing. Once cardboard baby box projects became widespread, however, and the Scottish Government commenced distribution of baby boxes to families of all new babies born in Scotland, familiarity with the ‘box to put the baby in’ increased. If the polypropylene box study was repeated in the UK now, participants would have something to compare them against and given their many advantages the polypropylene boxes may well prove popular.

## 5. Conclusions

The aim of the polypropylene box programme was to educate parents about hazardous infant sleeping arrangements, particularly sleeping with a baby in the presence of contraindications, and to provide those who needed one with a safer option for keeping their baby close at night. This intervention was welcomed by younger parents, particularly single primiparous mothers, but was less popular with older participants [29]. The purpose of the NHS-commercial partnership baby box schemes in England were less clear [9]; however, many were presented to parents and the public as a SIDS prevention or safer sleep promotion innovation from Finland. The cardboard baby boxes were primarily used as a day-time infant container—a place where babies could be laid in relative safety whether awake or asleep [18]. The polypropylene boxes also served this function and were considered more portable and hygienic, with the baby more easily visible to the caregiver.

In future, those designing box-type interventions should establish a clear purpose for issuing boxes to parents to put their babies in; for both interventions reviewed here the box itself was primarily a ‘hook’ to draw in parents and encourage them to engage with health-related education, and this may be their biggest benefit. However, the responses of parents to the boxes, and the ways in which they described using them suggests that the versatility of the polypropylene boxes for monitoring and supporting proximity with infants under a variety of circumstances day and night makes them preferable over cardboard boxes as infant sleep spaces.

Offering polypropylene boxes as one part of a SUDI prevention programme for families with infants at high-risk, particularly those lacking stable living arrangements, regularly spending the night in different locations, or for those caring for an infant alone may help them to meet their baby’s need for a clear, flat, safe sleep space. We also suggest that polypropylene boxes should be trialled as a potential way to facilitate ‘modified bed-sharing’ for infants born prematurely or of low birthweight, in order to facilitate night-time breastfeeding and responsive care.

## Figures and Tables

**Table 1:** Characteristics of evaluation participants.

Participant Characteristics	Polypropylene Box Scheme	Cardboard Box Scheme
*N*	79	77
Follow-up	68 (86%)	65 (89%)
Mean Maternal Age	21.4 years	no data
Primiparas	51 (75%)	no data
Partnered	44 (64%)	no data
Smokers	24 (35%)	no data
Post-compulsory education	2 (3%)	no data
Ethnicity = ‘White’	66 (97%)	no data

**Table 2:** Education re. box use reported by box recipients.

Education Type	Polypropylene Box Scheme (*n* = 68)	Cardboard Box Scheme (*n* = 65)
Received any information about safe box use	97.5%	95%
Written information	97.5%	54%
In-person discussion with health professional	90%	7%
Narrated video	82%	74%

## Data Availability

Data from the Let’s Talk About Sleep Study and the Baby Box Evaluation Study are available from the corresponding author on request.
